# Algorithms for Particle Detection in Complex Plasmas

**DOI:** 10.3390/jimaging5020030

**Published:** 2019-02-21

**Authors:** Daniel P. Mohr, Christina A. Knapek, Peter Huber, Erich Zaehringer

**Affiliations:** Deutsches Zentrum für Luft- und Raumfahrt e. V., Institut für Materialphysik im Weltraum, 82234 Wessling, Germany

**Keywords:** image processing, complex plasmas, blob detection, low-pass filter, Hanning amplitude filter, automatic threshold detection, Otsu’s method, image moments, geometric moments, particle tracking velocimetry (PTV), 07.05.Kf, 07.05.Pj, 52.27.Lw, 52.70.Nc

## Abstract

In complex plasmas, the behavior of freely floating micrometer sized particles is studied. The particles can be directly visualized and recorded by digital video cameras. To analyze the dynamics of single particles, reliable algorithms are required to accurately determine their positions to sub-pixel accuracy from the recorded images. Typically, a straightforward algorithm such as the moment method is used for this task. Here, we combine different variations of the moment method with common techniques for image pre- and post-processing (e.g., noise reduction and fitting), and we investigate the impact of the choice of threshold parameters, including an automatic threshold detection, on synthetic data with known attributes. The results quantitatively show that each algorithm and method has its own advantage, often depending on the problem at hand. This knowledge is applicable not only to complex plasmas, but useful for any kind of comparable image-based particle tracking, e.g., in the field of colloids or granular matter.

## 1. Introduction

Particle detection in digital images is a crucial first step in the analysis of many-particle systems in the case that individual particles can be detected by direct optical measurements. Efforts to optimize particle detection can be found in a wide range of fields: in biophysics, single particle tracking is used to study the motion of particles (e.g., proteins, molecules or viruses) involved in cell membrane and intracellular activities [1,2,3]. Particle detection and tracking from optical measurements is used in granular matter research [4,5], and in colloidal physics, where the dynamics of systems of nano- to micrometer sized particles can be investigated by analyzing single particle motion from direct video microscopy [6,7].

Complex plasmas [8,9,10] consist of micrometer sized particles injected into a low-temperature plasma composed of electrons, ions and neutral gas. The particles become charged in the plasma environment (mainly by the faster electrons) and acquire high negative charges of several thousand elementary charges. They interact with each other via the electrostatic force, and can form crystalline structures, but also liquid-like and gaseous systems. These particles are large enough to be visible to digital cameras with an appropriate optic, and provide an excellent opportunity to study fundamental dynamics on the kinetic level of individual particles. In contrast to colloids, where particles are embedded in a viscous medium and therefore over-damped, complex plasmas are rather underdamped, since the friction caused by the neutral gas is small. Therefore, dynamical processes happen fast enough for a reasonable observation time, but still the particle motion is slow enough due to the high mass to be resolved by modern cameras (e.g., the typical Einstein frequency of the particle oscillation lies below 100Hz). The particles are usually illuminated with a sheet of laser light, and the scattered light can be observed with digital cameras. Since the particle distances are large (with a magnitude of several hundreds of micrometers) due to the strong repulsive force between them, individual particles can be observed directly as mostly disjunct small groups of illuminated pixels on the camera sensor.

From those “blobs” of pixels, particle positions can be determined to sub-pixel accuracy—a necessity for the study of dynamics of single particles—with an adequately chosen algorithm.

By detecting individual particles, and tracing them through consecutive images (this is possible if the particle displacement between two images is small enough to allow for a unique assignment), velocities can be obtained. This method is called Particle Tracking Velocimetry (PTV), and has the advantage of more precise velocity measurements [11] in contrast to Particle Image Velocimetry (PIV) [12], where only spatially averaged velocity vectors are obtained, especially in particle clouds too dense for single particle detection.

Complex plasmas are three-dimensional systems, and recently the interest in 3D optical particle diagnostics is growing [13,14]. To triangulate the real position of a particle in 3D space, additional requirements are imposed on particle detection algorithms. Hence, we are also looking for algorithms for the detection of particles which are nearby each other on the image plane due to their overlapping motion in different distances to the image plane—when the illumination volume is thicker than the minimum interparticle distance. These algorithms can also be useful for particle tracking in systems with a high packing density.

With the methods presented in this paper, we show that the normally acquired accuracy can be exceeded without unnecessarily increasing the complexity of the procedure. This is accomplished by combining simple image pre- and post-processing procedures with an improved version of the commonly used algorithm for blob detection, and to some extent by applying automatic threshold detection.

Usually, straightforward and direct methods are used for blob detection [6,15,16], which is justified by the simple search feature and the typically low image noise. We show that this approach can be improved by generalizing it to blobs being not necessarily simply connected sets of pixels. Other more complex blob detection algorithms, such as SimpleBlobDetector [17] [SimpleBlobDetector] or MSER [18], did not turn out to be satisfactory in our case.

Though some of the techniques are well-known, a combination of them as well as an investigation of their individual influence on the accuracy of the particle detection was not performed elsewhere to such an extent, especially for the typical particle shapes obtained in complex plasma experiments (for example, Feng et al. [15] are mostly involved in examining one particular core algorithm without pre- and post-processing, while Ivanov and Melzer [16] investigated some methods for pre- and post-processing, but they do not combine the methods in the result).

Here, we not only investigate pre-processing, particle detection and post-processing in combination, but also take into account particle sizes and several kinds of image noise in our results. Additionally, we introduce Otsu binarization as an automated procedure. We also show that the choice of methods strongly depends on the image features (e.g., noise).

Preliminary results of our analysis were presented earlier [19], and some of the methods and algorithms presented in this publication were already used in practice by the authors [20,21].

The paper is organized as follows: After a description of the general approach in Section 2, Section 3 shows how the artificial images are generated to test the quality of the algorithms. In Section 4, Section 5 and Section 6 the different steps of image processing and particle detection are presented in more detail, followed by some examples in Section 7. Finally, in Section 8 the results are discussed.

## 2. General Approach

The process of obtaining particle coordinates from experimental data (images) can be divided into the following necessary steps:

**Image acquisition** Get the image from real world.
**Image processing** Prepare/enhance the image (e.g., by filtering).
**Blob detection** Identify particle positions.
**Postprocessing** Enhance found positions of particles (e.g., fitting).

Each step is an own field of research. In the following, they are explained in the depth necessary for this work.

### 2.1. Image Acquisition

Image acquisition is part of the experiment, and is only mentioned here for completeness, since the details of the experimental procedures go beyond the scope of this paper. To get good images, we need a proper illumination of the particles, a matched optical system, and an appropriate camera with an applicable storage system.

In this step, image noise is introduced. The sources are manifold, e.g., thermal behavior of the camera chip, noise of the involved electronics, defect pixels or radiation influencing the complete system. The noise causes uncertainties, which can be abstracted as additive white Gaussian noise and salt and pepper noise, superimposed on the pixels. Pixels can appear dark (“pepper”) or bright (“salt”), regardless of the exposure, e.g., due to errors in the pixel electronics. Bright pixels are easy to detect by taking dark-frame images (e.g., an image taken with covered lens), dark pixels can be detected with more effort by taking gray images. If a list of defective pixels is available, some cameras are able to correct these listed pixels by averaging the surrounding ones.

In the following, we assume a camera giving 8 Bit gray scale images.

### 2.2. Image Processing

Preparing the image is a task extremely dependent on the blob detection algorithm to be chosen for the next step, e.g., an algorithm using edge detection will not work well if the edges of the blob are destroyed by applying a smoothing filter. In that case, a sharpening filter would be preferable.

One particle can be seen approximately as a point source of light, and the point spread function describes what we can expect to see on the image sensor. The point spread function defines how an ideal point source will be mapped by a system of optical components. In the case of point-like particles, the Airy disc [22] gives a good approximation of this mapping.

**Figure 1:**
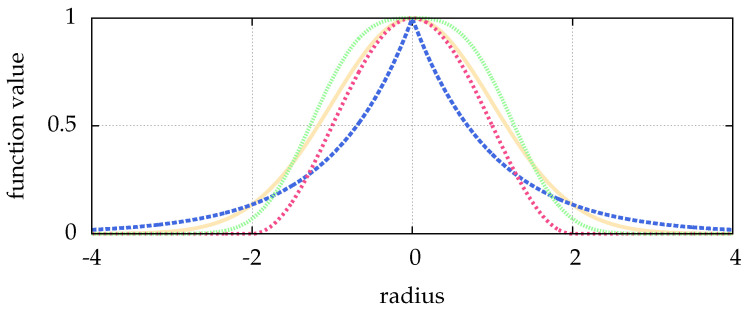
Shown are: the Hann function with N=5 in pink (■), and the generalized Gaussian point spread function with p=1 in blue (■), p=3 in green (■) and p=2 in yellow (■). The latter is identical to the normal distribution. The width is σ=1 for all cases. With different parameters *p*, the generalized Gaussian is able to mimic different particle shapes, which can e.g., result from defocused images.

Optical side lobes of the point spread function can be reduced by a Hanning amplitude filter (a convolution with the Hann function) [23]. The Hann function, visualized in Figure 1, with the parameter *N* for a point *r* is given by:

$$\begin{array}{c} \;w\left(r\right)=\left\{\begin{array}{cc} \frac{1}{2}\left(1-\cos\left(\frac{2\pi \left(r-\frac{N-1}{2}\right)}{N-1}\right)\right) & \mathrm{if}\;r\leq \frac{N-1}{2}\\ 0 & \mathrm{else}. \end{array}\right. \end{array}$$

The parameter *N* influences the width of the window. The Hanning amplitude filter is in principal a low-pass filter. This kind of filter passes signals with a spatial frequency less than the (user chosen) cutoff frequency, and can therefore reduce high-frequency image noise, e.g., Gaussian white noise.

This filter can easily be implemented by using template matching from the library opencv [17].

**Figure 2:**
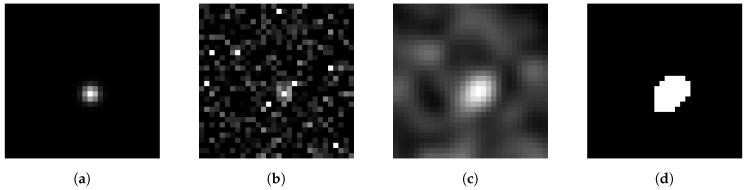
From left to right: (**a**) Image of a particle of size $\sigma_{x,y}=1$. In (**b**) high noise is added (Gaussian noise with SNR=5, and salt and pepper noise with a probability of 0.5%), (**c**) the noisy image is then filtered by a Hanning amplitude filter with N=5, (**d**) and finally the filtered image is clustered by Otsu’s method.

In Figure 2, an example shows the effect of a Hanning amplitude filter.

Of course it is in general a good idea to use combined low-pass and high-pass filtering. High-pass filtering does the opposite of a low-pass filter: it passes spatial frequencies above a cutoff and thus reduces image noise, such as large-scale intensity gradients. However, a high-pass filter can mask the behavior of a low-pass filter—e.g., the blurring of a low-pass filter would be reduced by a high-pass filter. Since we want to investigate the effect of specific filters, we do not want this masking. Usually, we do not observe low frequency noise in our images, and therefore omit high-pass filtering in this paper.

In general, it should be mentioned that Crocker and Grier [6] and Ivanov and Melzer [16] use a simple but effective filter, which behaves similar to a high-pass filter. They subtract a background calculated by a convolution with a boxcar kernel (moving average) from the image after low-pass filtering by a convolution with a Gaussian kernel.

### 2.3. Blob Detection

In the complex plasma community, a typical approach for blob detection is the moment method [15,16], which is a simplified version of the approach by Crocker and Grier [6] and [24]:

1. Find connected pixels brighter than a threshold (a particle).
2. Calculate center of every particle (position of a particle).

In the literature [15,16], connected pixels are assumed to be a set, which is simply connected. More general, we now define a set of pixels $P_{i}$ belonging to a particle as:

A particle is defined as a set of pixels with intensity values above a given threshold. This set can be just one pixel, or a group of pixels meeting certain constraints: the distance between a pixel, and all other candidates above the threshold, must be smaller than a user-given value, excluding all pixels with a greater distance. After identification of the pixels belonging to a particle, other constraints are possible, such as demanding a certain minimum number of pixels for a particle, or a certain minimum horizontal and/or vertical extent of the pixel blob. Another constraint could be the minimum density (the number of identified pixels divided by its rectangular envelope), or the minimum brightness density: the summed up intensity divided by the number of pixels.

A formal definition is given by:

$$\begin{array}{cc} P_{i}\subset & \left\{p:I\left(p\right)>I_{threshold}\right\} \end{array}$$

$$\begin{array}{cc} \mathrm{with}: & \left(\left|P_{i}\right|=1\right)\vee \left(\forall p_{j}\in P_{i}:\exists p_{k}\in P_{i}\setminus \left\{p_{j}\right\}:d\left(p_{j},p_{k}\right)<r\right)\\ & \forall p_{j}\in P_{i}:\forall p_{k}\in \left\{p:I\left(p\right)>I_{threshold}\right\}\setminus P_{i}:d\left(p_{j},p_{k}\right)\geq r\\ & \left|P_{i}\right|\geq m_{p}\\ & x_{d}\left(P_{i}\right):=\max\left\{x\left(p_{j}\right)-x\left(p_{k}\right):p_{j},p_{k}\in P_{i}\right\}\geq m_{x}\\ & y_{d}\left(P_{i}\right):=\max\left\{y\left(p_{j}\right)-y\left(p_{k}\right):p_{j},p_{k}\in P_{i}\right\}\geq m_{y}\\ & \frac{\left|P_{i}\right|}{x_{d}\left(P_{i}\right)\times y_{d}\left(P_{i}\right)}\geq m_{d}\\ & \frac{1}{\left|P_{i}\right|}\sum_{p\in P_{i}}^{}I(p)\geq m_{bd} \end{array}$$

Here, $P_{i}$ is a set of pixels and represents the particle with the number *i*, I(p) is the intensity of the pixel *p*, $I_{threshold}$ is the intensity of the threshold, $\left|P_{i}\right|$ denotes the cardinal number of $P_{i}$, $d(p_{j},p_{k})$ is the distance of the two pixels $p_{j}$ and $p_{k}$, *r* is a search radius, $m_{p}$ is the minimum number of pixels a particle needs to be composed of, x(p) is the *x* coordinate of *p*, y(p) is the *y* coordinate of *p*, $m_{x}$ is the minimum length in *x* direction in pixel, $m_{y}$ is the minimum length in *y* direction in pixel, $m_{d}$ is the minimum density of a particle (density being the total number of pixels weighted by the area of the smallest rectangle envelope of $P_{i}$), $m_{bd}$ is the minimum brightness density. The brightness density is defined as the sum of all intensity values of the pixels in $P_{i}$, weighted by the total number of pixels in the set $P_{i}$.

The parameter *r* allows to identify sets of pixels as a particle $P_{i}$ even if those pixels are not directly connected. For example, setting r=1 leads to a simply connected set as used in the mentioned literature, while setting $r=1.5>\sqrt{1^{2}+1^{2}}$ (assuming quadratic pixels with side length 1) allows pixels in $P_{i}$ to be connected only by a corner. For larger values of *r*, the pixels in the set $P_{i}$ do not need to be simply connected at all. This can be used for compensation of pepper noise or intensity jitter. In addition, to be recognized as separate particles, the shortest distance between the particle contours of two neighboring particles must be ≥r.

The center $\left(x_{c}\left(P_{i}\right),y_{c}\left(P_{i}\right)\right)$ can be calculated using the pixel positions and—as often done in the mentioned literature—the brightness of them:

(1) $$\begin{array}{cc} x_{c}\left(P_{i}\right)= & \frac{\sum_{p\in P_{i}}^{}x\left(p\right)\left(I\left(p\right)-I_{base}\right)}{\sum_{p\in P_{i}}^{}\left(I\left(p\right)-I_{base}\right)} \end{array}$$

(2) $$\begin{array}{cc} y_{c}\left(P_{i}\right)= & \frac{\sum_{p\in P_{i}}^{}y\left(p\right)\left(I\left(p\right)-I_{base}\right)}{\sum_{p\in P_{i}}^{}\left(I\left(p\right)-I_{base}\right)} \end{array}$$

Here, $I_{base}$ gives an offset. In Feng et al. [15] this offset is discussed and it is recommended to use $I_{base}=I_{threshold}$ to reduce the error.

Other blob detection algorithms (Bradski [17] [SimpleBlobDetector and MSER]) were tested, but proved to be unreliable and could only detect some of our largest particles. Since those algorithms increase the complexity and computation time without reaching the quality of our proposed blob detection method for the small particle images prevalent in complex plasmas, they were not investigated further.

### 2.4. Postprocessing

Since the blob detection is not an exact deconvolution, we are bound to have errors. To overcome this, we can fit a function modelling the particle shape (see Section 6) to the approximate particle coordinates as they were obtained from the blob detection. We now use the concept of a particle as an approximate point source of light, and the subsequent description of $P_{i}$ as a point spread function similar to the Airy disc [22]. The latter can be approximated by a Gaussian or a generalized Gaussian point spread function [25] (see (4) in Section 6), visualized in Figure 1.

In our procedure, we choose a generalized Gaussian point spread function and fit it to the approximate coordinates from the blob detection.

## 3. Simulated Images

To test our implementation we need well-defined, artificial images of particles. The images are modelled after real-world experimental images of complex plasmas recorded by optical cameras. Here, the use of artificial images with well-defined particle positions is crucial to be able to calculate the deviation of tracked position to real position and thus to quantify the quality of our algorithms.

The particles are represented by a bivariate normal distribution with a correlation of 0, the mean ${\left(\mu_{x},\mu_{y}\right)}^{T}$ match the center coordinate of the particle and the standard deviations $\sigma_{x},\sigma_{y}$ correspond to the particle size in the related coordinate direction:

(3) $$\begin{array}{c} \frac{1}{2\pi \sigma_{x}\sigma_{y}}\exp\left(\frac{-1}{2}\left(\frac{{\left(x-\mu_{x}\right)}^{2}}{\sigma_{x}^{2}}+\frac{{\left(y-\mu_{y}\right)}^{2}}{\sigma_{y}^{2}}\right)\right) \end{array}$$

In a real-life camera image, the brightness of a pixel is the integration over time and space, where the intervals for time and space are given by the exposure time and pixel size. Therefore, we integrate the intensity values over the size of one pixel:

$$\begin{array}{cc} & \int_{x_{1}}^{x_{2}}\int_{y_{1}}^{y_{2}}\frac{1}{2\pi \sigma_{x}\sigma_{y}}\exp\left(\frac{-1}{2}\left(\frac{{\left(x-\mu_{x}\right)}^{2}}{\sigma_{x}^{2}}+\frac{{\left(y-\mu_{y}\right)}^{2}}{\sigma_{y}^{2}}\right)\right)dydx\\ = & \frac{1}{2\pi \sigma_{x}\sigma_{y}}\left(\int_{x_{1}}^{x_{2}}\exp\left(\frac{-{\left(x-\mu_{x}\right)}^{2}}{2\sigma_{x}^{2}}\right)dx\right)\times \left(\int_{y_{1}}^{y_{2}}\exp\left(\frac{-{\left(y-\mu_{y}\right)}^{2}}{2\sigma_{y}^{2}}\right)dy\right)\\ = & \frac{1}{4}\left(\mathrm{erf}\left(\frac{x_{1}-\mu_{x}}{\sqrt{2}\sigma_{x}}\right)\;-\mathrm{erf}\left(\frac{x_{2}-\mu_{x}}{\sqrt{2}\sigma_{x}}\right)\;\right)\times \left(\mathrm{erf}\left(\frac{y_{1}-\mu_{y}}{\sqrt{2}\sigma_{y}}\right)\;-\mathrm{erf}\left(\frac{y_{2}-\mu_{y}}{\sqrt{2}\sigma_{y}}\right)\;\right) \end{array}$$

The constant factor $\frac{1}{4}$ can be ignored, because the image is rescaled to values between 0 and 1 in the end. This procedure is repeated for each pixel.

Furthermore, a particle may move during the exposure time with the constant velocity $v={\left(v_{x},v_{y}\right)}^{T}$ and has the coordinate μ(t) at the time t∈[0,1]:

$$\begin{array}{c} \mu \left(t\right)=\left(\begin{array}{c} \mu_{x}\left(t\right)\\ \mu_{y}\left(t\right) \end{array}\right)=\left(\begin{array}{c} v_{x}\cdot t+\left({\tilde{\mu }}_{x}-0.5v_{x}\right)\\ v_{y}\cdot t+\left({\tilde{\mu }}_{y}-0.5v_{y}\right) \end{array}\right) \end{array}$$

Again, the particles are represented by a bivariate normal distribution with a correleation of 0 as stated in (3), but with $\mu_{.}\left(t\right)$ instead of $\mu_{.}$

The integration over time and space yields:

$$\begin{array}{cc} & \int_{0}^{1}\int_{x_{1}}^{x_{2}}\int_{y_{1}}^{y_{2}}\frac{1}{2\pi \sigma_{x}\sigma_{y}}\exp\left(\frac{-1}{2}\cdot \left(\frac{{\left(x-\mu_{x}\left(t\right)\right)}^{2}}{\sigma_{x}^{2}}+\frac{{\left(y-\mu_{y}\left(t\right)\right)}^{2}}{\sigma_{y}^{2}}\right)\right)dydxdt\\ = & \frac{1}{2\pi \sigma_{x}\sigma_{y}}\int_{0}^{1}\left(\int_{x_{1}}^{x_{2}}\exp\left(\frac{-{\left(x-\mu_{x}\left(t\right)\right)}^{2}}{2\sigma_{x}^{2}}\right)dx\right)\left(\int_{y_{1}}^{y_{2}}\exp\left(\frac{-{\left(y-\mu_{y}\left(t\right)\right)}^{2}}{2\sigma_{y}^{2}}\right)dy\right)dt\\ = & \frac{1}{4}\int_{0}^{1}\left(\mathrm{erf}\left(\frac{x_{1}-\mu_{x}\left(t\right)}{\sqrt{2}\sigma_{x}}\right)\;-\mathrm{erf}\left(\frac{x_{2}-\mu_{x}\left(t\right)}{\sqrt{2}\sigma_{x}}\right)\;\right)\left(\mathrm{erf}\left(\frac{y_{1}-\mu_{y}\left(t\right)}{\sqrt{2}\sigma_{y}}\right)\;-\mathrm{erf}\left(\frac{y_{2}-\mu_{y}\left(t\right)}{\sqrt{2}\sigma_{y}}\right)\;\right)dt \end{array}$$

**Figure 3:**
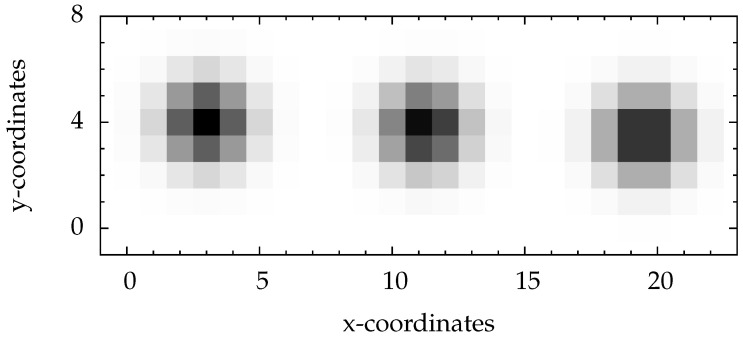
Simulated particles with $\sigma_{x}=1=\sigma_{y}$ without image noise. For better visibility the image is shown in inverted colors. The coordinates (3,4) of the left particle are centered in a pixel, the coordinates (19.5,3.5) of the right particle are exactly centered between two pixels, and the coordinates (11.25,3.8) of the middle one are chosen arbitrarily.

**Figure 4:**
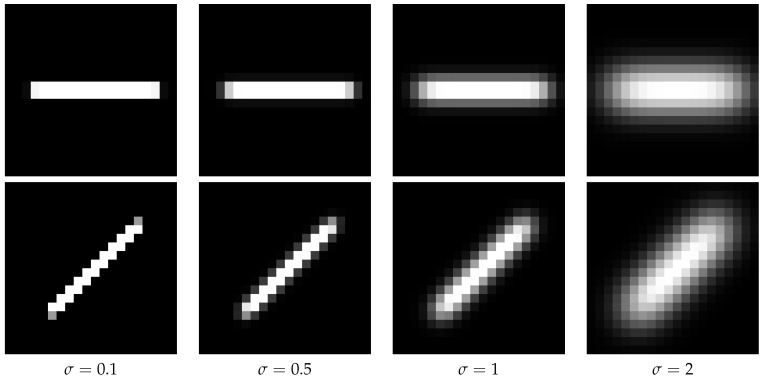
Different stripes of a particle resulting from different particle sizes and different angles of motion. The velocity of the illustrated particles is 15pixel/exposure. In the top row the angle is 0° and in the bottom row it is 45°. From left to right in the columns different particle sizes are presented. In all cases the *x* coordinate of the mean is centered in a pixel and the *y* coordinate of the mean is exactly centered between two pixels.

Examples for artificial particle images are given in Figure 3 and Figure 4. The figures also illustrate the impact of the given sub-pixel location of the particle center on the intensity distribution.

To be able to describe the strength of the noise by one single parameter, we create an additive white Gaussian noise (AWGN) with a mean of 0 and a standard deviation of 1. We can scale the noise to the image by a signal to noise ratio SNR with *B* a matrix representing the noise free image, $B_{noise}$ a matrix representing the noise and $B_{noisy}$ a matrix representing the image with noise:

$$\begin{array}{c} B_{noisy}=\max\left\{0,\min\left\{1,B+\frac{1}{\mathrm{SNR}\;}B_{noise}\right\}\right\} \end{array}$$

With this widely-used, simple noise model (e.g., it is often used in information theory [26,27]), we can create a noise which behaves roughly similar to the thermal noise of camera sensors. In Pitas [28] (pp. 43–44), this approach of cutting values is used to generate additive Laplacian noise (Because of a simple pseudo random number generator it was necessary to use a Laplacian instead of a Gaussian distribution in [28] (pp. 43–44)).

**Figure 5:**
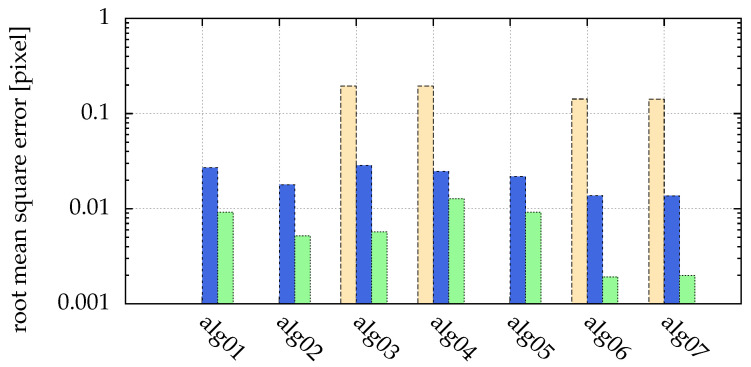
Comparison of different strengths of additive white Gaussian noise: yellow (■) bars for SNR=10, blue (■) for SNR=100 and green (■) for SNR=1000. Missing bars imply that not all particles where correctly detected (for SNR=5, this was the case for all algorithms). The ordinate shows the root mean square error of the distance between detected and real positions. There was no salt and pepper noise. The statistics/simulation was done with images containing 10,000 particles with $\sigma_{\left\{x,y\right\}}=1$.

Our simple SNR is consistent with the well-known Rose criterion (Rose [29] (p. 97)), which states that a SNR of at least 5 is necessary for a reliable detection. Due to this fact, Figure 5 does not show bars for SNR=5.

By setting pixel intensities to 0 or 1 with a given probability, we can add salt and pepper noise. This kind of noise simulates defective pixels usually present on typical camera sensors.

Though the occurrence of excessive salt and pepper noise in an experimental setup should normally lead to an exchange of hardware, there are situations in which this is not an option. Good examples are experimental instruments in remote locations not accessible to technicians, e.g., satellites or sealed experimental devices on a space station, such as complex plasma microgravity research facilities (PK-3 Plus [30], PK-4 [31]). Here, cameras are subjected to higher levels of radiation, and pixel deterioration, causing salt and pepper noise, becomes an issue. To still obtain good scientific results over an extended period of time, one needs to handle such noise sources adequately during data analysis as long as it is feasible.

## 4. Preprocessing (Image Processing)

Image preprocessing is not restricted to the use of general filters preserving the brightness distribution of particles, but can be extended to procedures for e.g., threshold detection, especially with regard to the requirements of the moment methods.

In the first step, the moment method needs a separation of the pixels belonging to particles, and pixels composing the background. Since our images represent particles illuminated with a laser, we can assume to have a bi-modal histogram.

**Figure 6:**
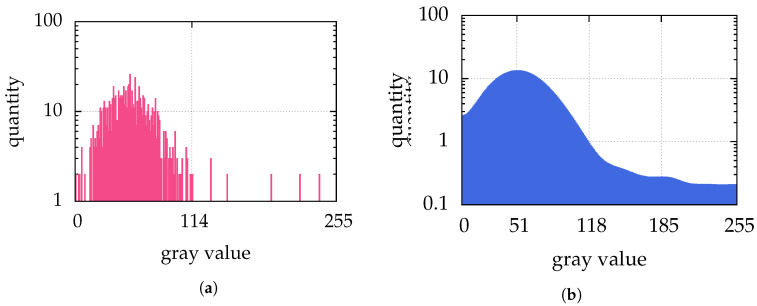
(**a**) shows the histogram of Figure 2c and (**b**) shows the final histogram of the intermodes thresholding iteration applied to the histogram of Figure 2c. The Otsu’s method finds a threshold for binarization of 114 in Figure 2c. The intermodes thresholding smoothes the histogram of Figure 2c until there are the two local maxima 51 and 185, which leads to a threshold of 118. Because the image Figure 2c has no gray values between 115 and 119, there is no difference in the binarization of Otsu’s method and intermodes thresholding. Therefore, for both methods, the binarization of Figure 2c is shown in Figure 2d.

This can be clustered for example by Otsu’s method [32]. This method separates the histogram of the image in 2 classes—below and above the threshold—with the goal to minimize the variances of both classes. This leads to a maximal interclass variance. The image is then binarized according to the classes—pixels of one class are usually shown as white, and those belonging to the other class as black. An example is shown in Figure 2d, which is the binarization of Figure 2c. The histogram of Figure 2c with the threshold found by Otsu’s method is shown in Figure 6a.

There are other thresholding techniques available (for an overview see e.g., Sezgin and Sankur [33]). We use Otsu’s method since it is in the top ten ranking of Sezgin and Sankur [33], one of the most referenced (therefore well-known), and implemented e.g., in opencv [17].

Furthermore, a quick visual check of our example images with the tool ImageJ [34] shows that most available other techniques lead to erroneous binarizations, with background pixels becoming falsely detected as signals and set to white.

We analyzed one of the more promising methods further: the intermodes thresholding of Prewitt and Mendelsohn [35] (e.g., implemented in ImageJ [34]) shows a similar detection rate, such as with Otsu’s method. It smoothes the histogram by running averages of a window size of 3 bins until there are exactly two local maxima (e.g., Figure 6b). The threshold is then the arithmetic mean of those. However, only in the example of particle separation (Section 7.2) the intermodes thresholding performs superior to Otsu’s method, because the intermodes thresholding chooses a higher threshold than Otsu’s method. The higher threshold is chosen in all our examples, and the reason for this is simple and shows also the drawback of intermodes thresholding: dominant peaks in the histogram—such as the peak at 1 in our perfectly “illuminated” artificial images—are detected as one maximum and shift the average towards the maximum brightness value. Nonetheless, the performance of such a simple approach is excellent.

In Figure 2, an image is shown representing the clustering by Otsu’s method. For all further steps of calculating the particle center only the threshold value detected by Otsu’s method is used, not the binarized image itself. This means that in the first step of the moment method the threshold is used to identify “white” pixels belonging to a particle, while in the second step the position is calculated with the brightness values of the original image.

A simple approach is gamma adjustment: Every pixel value is raised to the power of the parameter γ. For γ>1 this increases the contrast for bright objects.

Different algorithms are compared with respect to different signal to noise ratios in Figure 5:

**alg01**  moment method (Section 2.3)
**alg02**  alg01 with $I_{base}=I_{threshold}$
**alg03**  alg02 preprocessed by a Hanning filter (N=5)
**alg04**  alg03 with the threshold automatically detected by Otsu’s method
**alg05**  alg04 with gamma adjustment with γ=3
**alg06**  alg01 preprocessed by a Hanning filter (N=5) and fitted by a generalized Gaussian (Section 6)
**alg07**  alg06 with the threshold automatically detected by Otsu’s method

**Figure 7:**
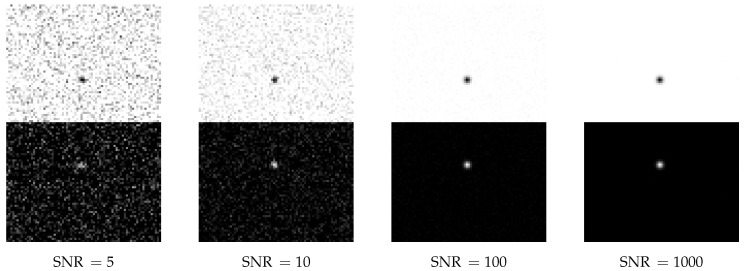
For 4 different signal to noise ratios example images are given, which were used in Figure 5. Each example shows 2 particles—one in the upper and and one in the lower half. The lower halves of the figures show the original images, while the upper halves are shown in inverted colors for better visibility.

Examples of single noisy particle images are shown in Figure 7. For a SNR of 5, not all of the 10,000 particles could be detected by any of the algorithms. While particles in the example images Figure 7 are easy to identify for human eyes, the algorithms are more sensitive to the noise.

Comparing alg01 and alg03 in Figure 5, we can see that the Hanning filter used in alg03 leads to a better detection rate in the case of high noise.

Feng et al. [15] recommend using $I_{base}=I_{threshold}$ in the moment method to reduce uncertainties in the found particle positions. alg02 uses this method, and Figure 5 shows that indeed the error can be reduced in comparison with the pure moment method in alg01.

**Figure 8:**
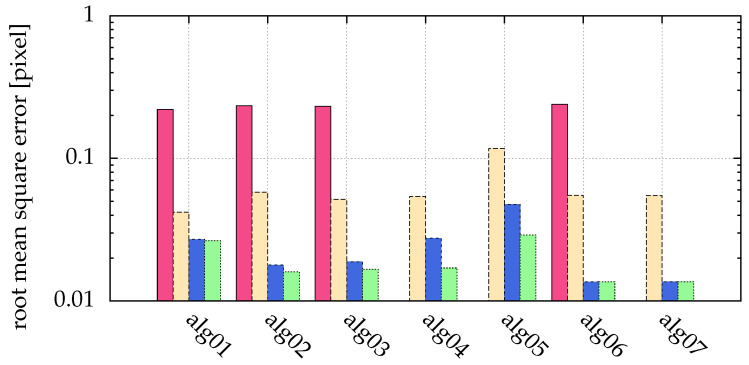
Comparison of different particle sizes: pink (■) bars for $\sigma_{\left\{x,y\right\}}=0.1$, yellow (■) for $\sigma_{\left\{x,y\right\}}=0.5$, blue (■) for $\sigma_{\left\{x,y\right\}}=1$ and green (■) for $\sigma_{\left\{x,y\right\}}=2$. Missing bars imply that not all particles where correctly detected. The ordinate shows the root mean square error of the distance between detected and real positions. There was a low Gaussian noise with a SNR=100, and no salt and pepper noise. The statistics/simulation was done with images containing 10,000 particles.

**Figure 9:**
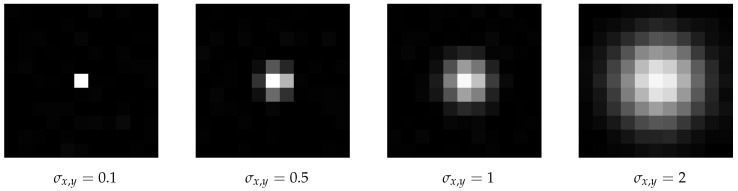
Example images of the different particle sizes used in Figure 8.

**Figure 10:**
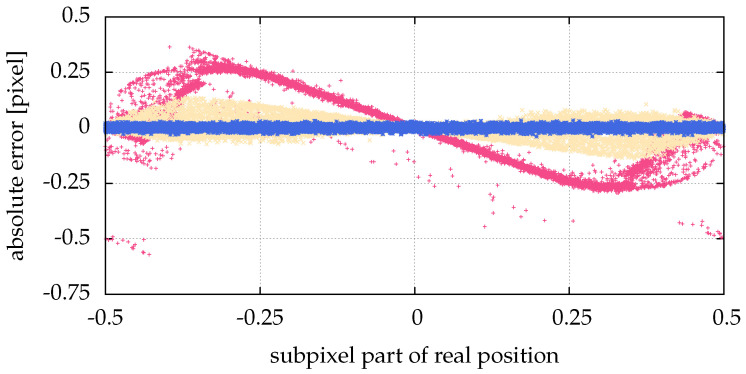
Comparison of different particle sizes: pink (■) points for $\sigma_{\left\{x,y\right\}}=0.1$, yellow (■) for $\sigma_{\left\{x,y\right\}}=0.5$ and blue (■) for $\sigma_{\left\{x,y\right\}}=1$. For $\sigma_{\left\{x,y\right\}}=2$, the same visual result was obtained as for $\sigma_{\left\{x,y\right\}}=1$. There was a low Gaussian noise added, with a SNR=100, and no salt and pepper noise. The statistic/simulation was done with images containing 10,000 particles. The abscissa shows the sub-pixel coordinate of the real positions, whereas the ordinate shows the respective absolute error of the positions calculated with alg06.

However, this is not true for small particles ($\sigma_{\left\{x,y\right\}}\in \left\{0.1,0.5\right\}$), as shown in Figure 8. While Feng et al. [15] explain, why an inappropriately chosen threshold leads to pixel locking (freezing of a detected particle coordinate while changing the original particle coordinate), here we see that another reason for pixel locking can be missing information, such as particles consisting of not enough pixels, as seen in Figure 9. The origin of this error is not the algorithm, but the measurement. The measurement conditions, such as insufficient illumination or resolution, are inadequate. Figure 10 illustrates the influence of the particle size on alg06. For particle sizes $\sigma_{\left\{x,y\right\}}\in \left\{0.1,0.5\right\}$, the positions calculated by alg06 are not statistically fluctuating around the real position. Instead, there is a systematic deviation depending on the real position—a similar behavior can be observed for all presented algorithms. For example, a particle with $\sigma_{\left\{x,y\right\}}=0.1$ consists of more than one pixel only, if the absolute value of the chosen sub-pixel coordinate is greater than 0.25 (cf. Figure 9). Therefore, for a coordinate with an absolute value of the chosen sub-pixel coordinate of less than 0.25, any algorithm can find just that one pixel, and consequently only detect the exact coordinate of it, which yields 0 as the sub-pixel coordinate. In a more general sense, if the real particle coordinate is changed in a neighborhood (open set containing the real particle coordinate) and the found particle coordinate does not change (is locked), this is called pixel locking.

The clustering by Otsu’s method used in alg04 and alg07 performs well. Only for very small particles, in the example given by Figure 8 and visualized in Figure 9, a stable detection is not possible. Increasing gamma (alg05) does slightly improve the accuracy of alg04, but not all particles can be detected any more. Comparing alg02 and alg04 shows that Otsu’s method does not choose the best threshold. However, as an automatic procedure processing all available pixel values, it can reduce human errors in the process of choosing the threshold.

## 5. Moment Method (Blob Detection)

**Figure 11:**
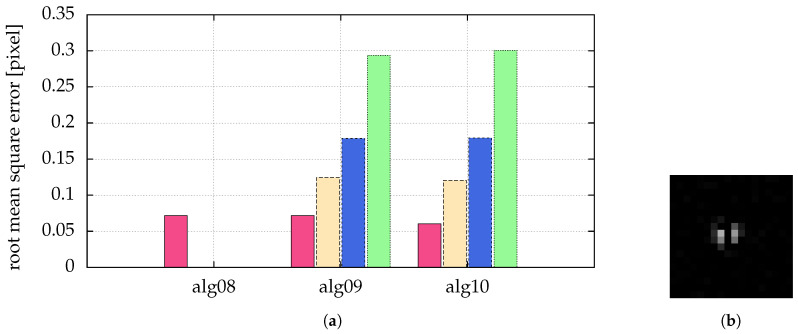
(**a**) Comparison of different strength of pepper noise: pink (■) bars for a probability of pepper noise of 1%, yellow (■) for 5%, blue (■) for 10% and green (■) for 20%. Missing bars imply that not all particles where correctly detected. The ordinate shows the root mean square error of the distance between detected and real positions. The Gaussian noise was combined with a SNR=100. The statistics/simulation was done with images containing 10,000 particles with $\sigma_{\left\{x,y\right\}}=1$. (**b**) The right image shows a single particle separated by pepper noise with a probability of 20%.

In Figure 11, different algorithms are compared with respect to different probabilities of pepper noise:

**alg08**  alg01 with a search radius r=1 (moment method with particles being only single connected sets, similar to [15,16])
**alg09**  alg01 with a search radius r>1
**alg10**  alg01 preprocessed by a Hanning filter (N=5)

We can see that for high pepper noise alg08 is not able to detect all particles correctly—it finds too many, because some particles are split in two by the pepper noise.

**Figure 12:**
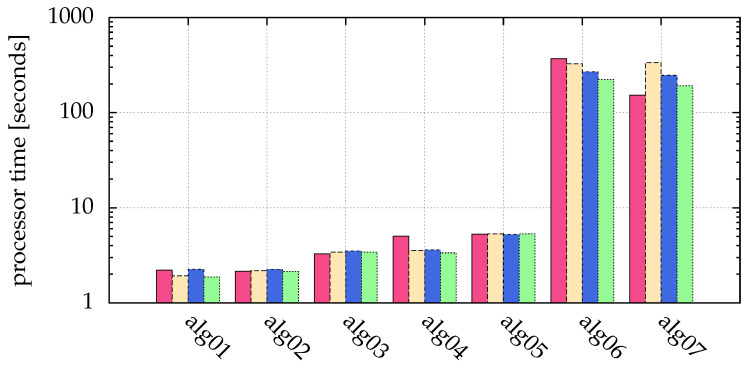
Comparison of used processor time of the simulations of Figure 5: pink (■) bars for SNR=5, yellow (■) for SNR=10, blue (■) for SNR=100 and green (■) for SNR=1000. The ordinate shows the measured processor time in seconds on an Intel Xeon processor E5-2643v3. The statistics/simulation was done with images containing 10,000 particles with $\sigma_{\left\{x,y\right\}}=1$.

Using alg09, the generalized moment method described in Section 2.3, we are able to detect all particles correctly. The same holds for the Hanning filter in alg10. The quality of the latter is comparable to the generalized moment method. The only draw-back is the larger computing time of alg10 (see Figure 12, comparison of used processor times of alg02 and alg03).

The moment method described in Section 2.3 calculates the center by averaging the weighted intensities of image pixels in (1) and (2). The basic concept

$$\begin{array}{ccc} x_{c}\left(P_{i}\right)= & \frac{\sum_{p\in P_{i}}^{}x(p)I(p)}{\sum_{p\in P_{i}}^{}I(p)};\;y_{c}\left(P_{i}\right)= & \frac{\sum_{p\in P_{i}}^{}y(p)I(p)}{\sum_{p\in P_{i}}^{}I(p)} \end{array}$$

without the enhancement of Feng et al. [15] is just calculating the centroid or the center of mass by using raw moments (also known as spatial or geometric moments) [36].

In general, image moments [36] describe the pixels of an image or a subset. They are used in image recognition and allows assigning properties to the set of pixels.

The raw moments of an image are defined as:

$$\begin{array}{cc} m_{i,j}= & \sum_{x}^{}x^{i}y^{j}I\left(x,y\right)\mathrm{for}\;i,j\in N_{0} \end{array}$$

Here, x,y are the coordinate of the pixel with the intensity I(x,y).

For a particle described by the pixels $P_{i}$ we get:

$$\begin{array}{cc} {\left(x_{c}\left(P_{i}\right),y_{c}\left(P_{i}\right)\right)}^{T}= & {\left(\frac{m_{1,0}}{m_{0,0}},\frac{m_{0,1}}{m_{0,0}}\right)}^{T} \end{array}$$

Consider a stripe of given length in the image as a result of a particle moving during the exposure time (see Figure 4). This can be interpreted as a circular disk (the still particle image) moving along a line segment of length *l*. This yields a set of pixels *J* belonging to the area A which was covered by the moving disk. The area *A* can be calculated from the raw moment $m_{0,0}$ and the intensity sum *s* over all pixels as $A:=\frac{m_{0,0}}{s}$. The assumption is that the disk only moves in a straight line. $l_{m}:=\max_{x_{1},x_{2}\in J}{\left|\left|x_{1}-x_{2}\right|\right|}_{2}$ is the maximal distance between two points out of the set. With *r* the radius of the disk we get 2 equations to calculate *l* from the image:

$$\begin{array}{cc} A= & 2\cdot \frac{r^{2}\pi }{2}+l\cdot 2r\\ l_{m}= & l+2r \end{array}$$

Now we can solve these equations for the length and get:

$$\begin{array}{cc} l= & \frac{-b-\sqrt{b^{2}-4ac}}{2a}\mathrm{with}\;a:=\frac{\pi }{4}-1,\;b:=\left(1-\frac{\pi }{2}\right)l_{m}\mathrm{and}c:=\frac{\pi }{4}l_{m}^{2}-A \end{array}$$

Other properties are derived from the central moments [36]:

$$\begin{array}{cc} \mu_{i,j}= & \sum_{x}^{}\sum_{y}^{}{\left(x-\bar{x}\right)}^{i}{\left(y-\bar{y}\right)}^{j}I\left(x,y\right)\mathrm{with}\bar{x}:=\frac{m_{1,0}}{m_{0,0}}\mathrm{and}\frac{m_{0,1}}{m_{0,0}} \end{array}$$

Stojmenović and Žunić [37] described how to use central moments to calculate an angle α of the point set, which corresponds to the direction of motion of the particle with respect to a chosen axis:

$$\begin{array}{cc} \alpha = & \frac{1}{2}\arctan\left(\frac{2\mu_{1,1}}{\mu_{2,0}-\mu_{0,2}}\right) \end{array}$$

As a practical example, in the experiment PK-4 [31] flowing complex plasmas are investigated, which leads to the above described feature of particles as stripes. To obtain particle flow velocities, the introduced procedures for calculating the angle and the length of the stripes present an elegant solution.

Weber et al. [38] showed that this problem can successfully considered by an exhaustive search using template matching. They generated different templates depending on possible lengths and angles, and compared these templates by template matching [17] using mutual information to measure the similarity between the template and every possible position of it in the image. The mutual information compares the entropy of the template with the clipping of the image.

**Figure 13:**
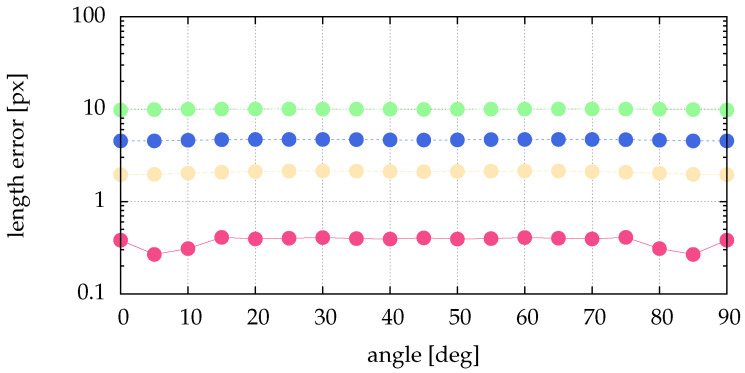
Comparison of length errors for different angles of the stripe and different particle sizes: pink (■) for σ=0.1, yellow (■) for σ=0.5, blue (■) for σ=1 and green (■) for σ=2. To overcome the dependence on the subpixel position shown in Figure 10 we averaged over 100 equally destributed subpixel positions. The length was varied between 1 px and 21 px in 11 equal steps. This means, every plotted length error is an average of 1100 analyzed artificial stripes. The scale of the ordinate is logarithmic.

**Figure 14:**
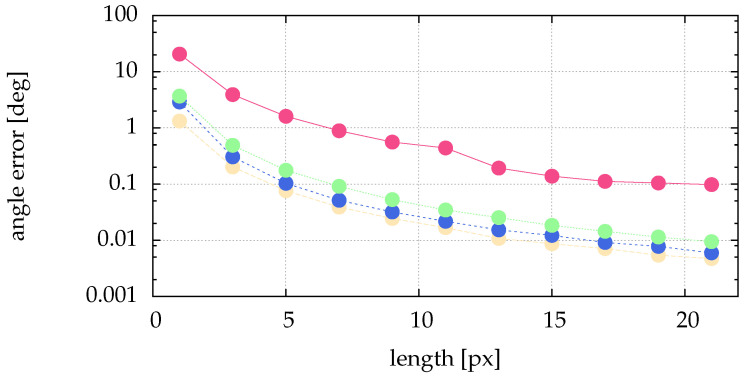
Comparison of angle errors for different lengths of the stripe and different particle sizes: pink (■) for σ=0.1, yellow (■) for σ=0.5, blue (■) for σ=1 and green (■) for σ=2. The angle was varied between 0° and 90° in 19 equal steps. To overcome the dependence on the subpixel position shown in Figure 10 we averaged over 100 equally destributed subpixel positions. This means, every plotted length error is an average of 1900 analyzed artificial stripes. The scale of the ordinate is logarithmic.

The above presented method of calculating the angle and the length directly from the image data needs much less compute power. To show the performance regarding determining angle and length, we simulate data and compare the calculated quantities with the simulated ones. From Figure 4 it is obvious that the preprocessing of Section 2.2 and Section 4 perform similar for stripes. Therefore, the analysis of the impact of image noise is not repeated here. Figure 13 shows no dependence of the length error on the angle. For narrow stripes (small particle size given by σ) we get an acceptable and reasonable length error. Figure 14 shows the angle error for different lengths. As expected, for longer stripes smaller errors result. Already for a stripe of the length 5 px the angle error becomes very small.

## 6. Fitting (Postprocessing)

Given approximate coordinates from the blob detection of the last Section 5, we can try to enhance them by fitting a generalized Gaussian point spread function, which is visualized in Figure 1 and given as:

(4) $$\begin{array}{c} \frac{p^{1-\frac{1}{p}}}{2\sigma \Gamma \left(\frac{1}{p}\right)}\times \exp\left(\frac{-{\sqrt{x^{2}+y^{2}}}^{p}}{p\sigma^{p}}\right) \end{array}$$

The fit is performed locally to every single particle. Therefore, we split the given image in non-overlapping squares with an approximated particle coordinate located in the center of the square. Every square is chosen with a maximal side length under the given restrictions.

Initially, the distance *d* of two particles *i* and *j* is defined as:

$$\begin{array}{c} d\left(\left(\begin{array}{c} x_{i}\\ y_{i} \end{array}\right),\left(\begin{array}{c} x_{j}\\ y_{j} \end{array}\right)\right):=\max\left\{|x_{i}-x_{j}\left|,\right|y_{i}-y_{j}|\right\} \end{array}$$

Then, for a given particle coordinate $p_{0}:={\left(x_{0},y_{0}\right)}^{T}$ the closest particle $p_{1}:={\left(x_{1},y_{1}\right)}^{T}$ is found as:

$$\begin{array}{c} \forall i:d\left(\left(\begin{array}{c} x_{i}\\ y_{i} \end{array}\right),p_{0}\right)\geq d\left(p_{1},p_{0}\right) \end{array}$$

With $\delta :=\frac{1}{2}d\left({\left(x_{1},y_{1}\right)}^{T},{\left(x_{0},y_{0}\right)}^{T}\right)$ and $\left\lfloor .\right\rfloor$, $\left\lceil .\right\rceil$ the floor and ceiling functions (In practice, this should not be a mapping to integers Z), but to image coordinates—a subset of non-negative integers $N_{0}$. we get the vertices of the square as:

$$\begin{array}{c} \left\{\left\lceil x_{0}-\delta \right\rceil ,\left\lfloor x_{0}+\delta \right\rfloor ,\left\lceil y_{0}-\delta \right\rceil ,\left\lfloor y_{1}+\delta \right\rfloor \right\} \end{array}$$

**Figure 15:**
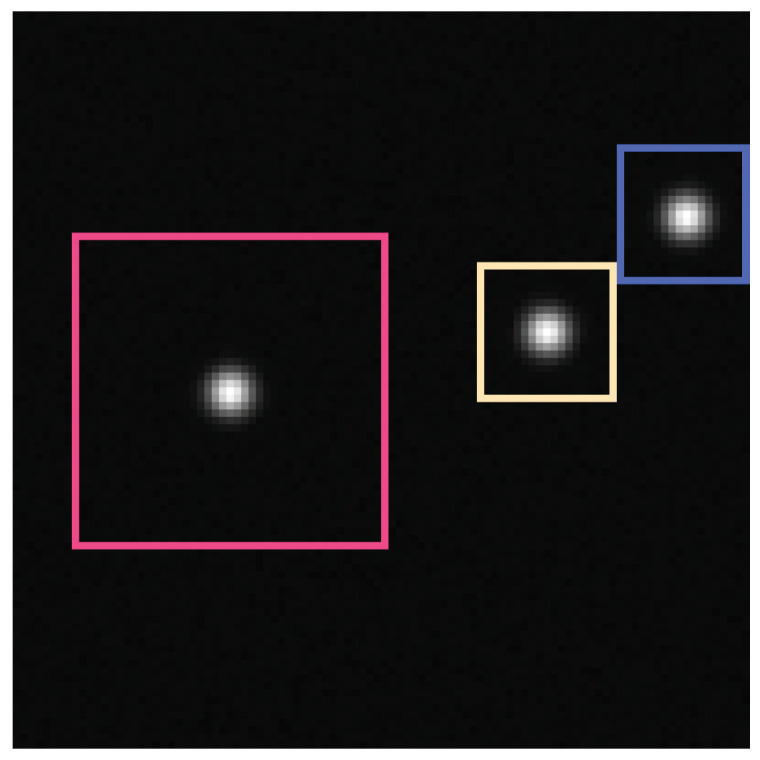
To perform the local fitting to every single particle, we have to split the given image in non-overlapping squares. Here, an example is given with 3 particles. This leads to the 3 marked rectangles—the colored (pink (■), yellow (■) and blue (■)) pixels belong to the rectangles. The sizes of the rectangles are: pink 18 × 19 ${\mathrm{px}}^{2}$, yellow 19 × 19 ${\mathrm{px}}^{2}$ and blue 43 × 43 ${\mathrm{px}}^{2}$.

The resulting areas are visualized in Figure 15 for 3 particles.

Now we generate separate problems for every square or particle. Let the given image be a matrix $I=\left(I_{i,j}\right)$. Here, we use the original image and not the prefiltered one.

An artificial image can be created with (4) as $A\left(x,\sigma ,p\right)=\left(A_{i,j}\left(x,\sigma ,p\right)\right)$ with the particle coordinate $x\in R^{2}$ and σ, *p* the parameters for the generalized Gaussian point spread function. With *b* the averaged brightness of the background, this results in the optimization problem:

$$\begin{array}{c} \min_{x,\sigma ,p,b}\sum_{i,j}^{}{\left(I_{i,j}-A_{i,j}\left(x,\sigma ,p\right)-b\right)}^{2} \end{array}$$

For solving this optimization problem we use the algorithm L-BFGS-B [39,40,41], implemented in the python module/library SciPy [42].

The gradient of the objective function is calculated numerically by a symmetric difference quotient if possible (e.g., on the boundary of the feasible solutions we cannot calculate a symmetric difference quotient).

In Figure 5, different algorithms were compared with respect to different signal to noise ratios, including those with fitting. Additionally, in Figure 12 the processor time used for the simulation is given. It is obvious that a small improvement by fitting a generalized Gaussian (alg06 and alg07) leads to a large calculation time (Figure 12 shows a factor of 60 to 144).

The improved detection rate by Hann filtering (e.g., alg03), and the automatically chosen threshold by Otsu’s method (e.g., alg04) each lead to a larger error, as demonstrated in Figure 8 and Figure 5. This can be corrected by fitting (e.g., alg06 and alg07).

As described, to do the fit locally we have to split the image in disjunct areas and do the fit in every single area. If not every pixel is assigned to an area, this could lead to loss of information. In addition, it is not clear that all pixels holding information for a particle are in the chosen area around this particle (e.g., 2 particles with a short distance on the image plane)—this would lead to loss of information. In contrast, fitting all particles in one image simultaneously uses all information. This assumption leads to a high dimensional optimization problem. In our implementation with the algorithm L-BFGS-B, this problem could not always be solved successfully. If it was successful, the result was sometimes slightly better than fitting every individual particle, but at the cost of a considerably increased computing time: it was about 100 times higher for 100 particles, and about 700 times higher for 1000 particles.

## 7. Examples

### 7.1. Velocity

In this section we will regard the velocity in images; this is the velocity of a particle in the image plane—e.g., a 2D mapping of a real 3D motion.

Let us assume a sequence of images with a temporal distance of dt=10 ms between 2 consecutive images (equivalent to a frame rate of 100 images per seconds), with particles modeled as a Gaussian with σ=1pixel and a SNR=100.

In our simulations (see Figure 5 and Figure 8), the presented algorithms alg06 and alg07 yield a root mean square error of about 0.014pixels (or better). Assuming a distribution around 0, the root mean square is the standard deviation.

When calculating a particle velocity from 2 consecutive particle positions, each subject to the same uncertainties, error propagation leads to an error of (2×0.014)/dt [px/s] in the velocity.

As an example, for a 4 megapixel camera (2048×2048 ${\mathrm{px}}^{2}$) with a field of view of 4cm by 4cm this leads to an uncertainty in the velocity of 0.056mm/s.

In Table 1, a few examples are given. We neglect here that changing the spatial resolution also changes the size of a particle on the image sensor. Otherwise, the error for the resolution of 0.005mm/px would be reduced dramatically.

With the last example representing a 4 megapixel camera (2048×2048 ${\mathrm{px}}^{2}$) at 80 frames per seconds with a field of view of 11mm by 11mm we would be able to measure the velocity of particles (⌀=9.19 μm, $\rho =1.51\;{g/\mathrm{cm}}^{3}$) at room temperature, which would be about 0.08mm/s. Experiments with a crystalline 2D complex plasma, and a comparable spatial camera resolution, were analyzed with the presented alg01 by Knapek et al. [20], yielding reasonable particle kinetic energies.

Knowing the uncertainties, especially for particle velocity calculation, should not be underestimated: Gaussian noise leads to a artificial Gaussian velocity distribution, which can easily mask a Maxwellian velocity distribution. However, it is not possible to separate the artificial distribution from the velocity distribution, since they are convoluted (see e.g., Knapek [43] (Chapter 7); here, as well as in other publications [20,44], the applicability of the presented algorithm alg01 to real-world data is demonstrated in more detail). Therefore, it is of high importance to know the limit of resolvable particle motion (depending on particle size, SNR and the algorithms) for a specific experiment before interpreting the results.

### 7.2. Particle Separation

**Figure 16:**
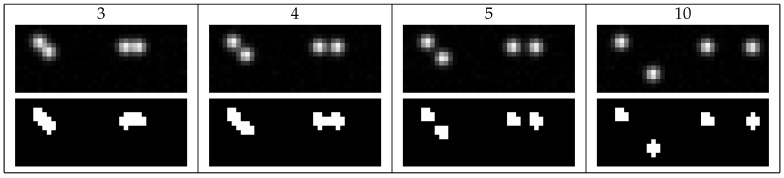
Different particle distances: In the first row, the minimal particle distances are given in pixel. In the second row, example images are given. In the last row, these example images are prefiltered by Otsu’s method as described in alg04.

Here, we assume two particles which are nearby each other on the image plane (see Figure 16), e.g., due to their overlapping motion in different distances to the image plane. Furthermore, we assume both particles have the same size on the image, e.g., an uniform illumination, a good enough depth of field and same particle size and texture.

We neglect the Gaussian beam profile of the laser in the simulation. This profile might give additional information about the depth of a particle: the pixel intensity values would reflect the (ambiguous) position of the particle within the spatial extent of the laser beam, and could be used for a relative depth evaluation between particles, but we do not use this kind of information in the presented algorithms.

Now we can use all algorithms introduced in Section 4. Since we want to separate both particles, we do not want to detect particle pictures as shown in Figure 11 (right) as one particle. Also, we cannot use a too large Hanning amplitude filter, because it would wash-out distinctive edges of nearby particles. Therefore, we have to demand a good SNR to avoid the necessity of preprocessing. This is usually available in typical images of complex plasmas obtained with a laser filter which suppresses background illumination (e.g., from the plasma glow).

We now introduce additional algorithms similar to alg03 and alg06:

**alg11**  alg02 with a large threshold of intensity 190 and a search radius r=1
**alg12**  alg11 with fitting a generalized Gaussian (Section 6)

**Figure 17:**
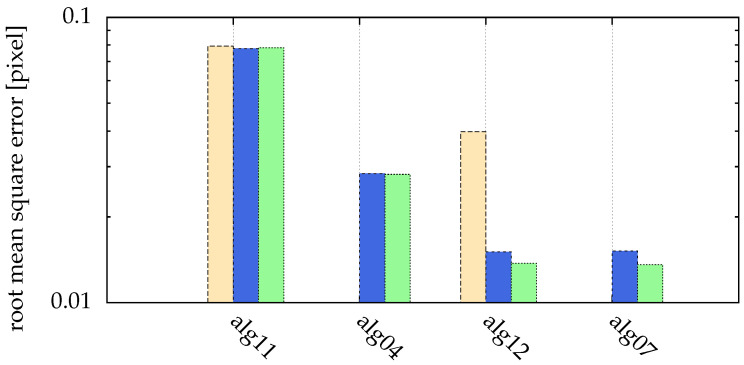
Comparison of different particle distances: yellow (■) for minimal particle distance of 4 pixel, blue (■) for minimal particle distance of 5 pixel and green (■) for minimal particle distance of 10 pixel. Missing bars imply that not all particles where correctly detected (for a distance of 3 pixel, this was the case for all algorithms). The ordinate shows the root mean square error of the distance between detected and real positions. The statistics/simulation was done with images containing 10,000 particles with $\sigma_{\left\{x,y\right\}}=1$ and SNR=100.

In Figure 17, different particle distances, as visualized in Figure 16, are compared. Here, for all presented algorithms a search radius of r=1 was used. Particles with distances of 3 pixels or less could not be separated. The moment method (alg11) described in Section 2.3 with a large threshold of 130 is able to separate particles with $\sigma_{\left\{x,y\right\}}=1$ with a distance of only 4 pixels. Choosing the threshold automatically (for minimal particle distances of 3, 4, 5 and 10 pixels, the threshold was 78, 71, 73 and 73, respectively) with alg04 separates these particles down to a distance of 5 pixels. In both cases, postprocessing the images by fitting (alg12 and alg07) reduced the uncertainties.

It may be possible to choose the objective function value of the optimum as an indicator for the success of a separation. For sure, this is not true for global fitting, since a few particles not separated influence the objective function value only marginally. If the indicator implies an inadequate separation, one could change the initial approach (e.g., preprocessing) to enhance the separation and retry the fitting. Since already the local fitting approach is an expensive operation, doing this repeatedly until the separation is satisfying is even more expensive.

The possibility to separate close-by particles can prove helpful in 3D diagnostics, e.g., for the analysis of data taken with a stereoscopic setup (several cameras viewing the same volume from different angles). Particles located close to each other on the image plane are typical features for this kind of diagnostics, and algorithms are needed to reliably detect particles in each of the camera views as the basis for a subsequent triangulation [45].

## 8. Discussion

In this paper, we presented a comparison of several methods and algorithms for particle detection from images. The methods and algorithms were tested on artificial images simulating data as they are obtained in complex plasma experiments, including realistic image noise (additive white Gaussian noise, salt and pepper noise). To increase the statistical significance, images with a large number of particles (10,000) were analyzed. The proposed procedure for particle tracking consists of three major steps: image processing, blob detection and postprocessing.

In Section 4, we show that using a Hanning filter to remove Gaussian noise during image processing results in a better detection rate in the presence of high noise, whereas the accuracy of the found positions is slightly reduced (Figure 5).

For images consisting of features (the particles) and a background (noise), the choice of a good threshold is important during image processing. With Otsu’s method (used in alg04, alg07), we introduce this concept of automatic thresholding for particle detection in complex plasma for the first time (Section 4). Other automatic thresholding techniques were tested, but did not prove to be suitable. The clustering by Otsu’s method performs very well (Figure 5), yielding almost the same results as the manually chosen threshold for all but the smallest particle sizes (Figure 8). On the one hand, choosing the right threshold value is not an easy task, and an automatic method can dramatically reduce human errors. On the other hand, an automatism prohibits using expert knowledge of the user in special circumstances, e.g., for the task of particle separation (Section 7.2).

In Section 2.3 we introduce an improved algorithm for blob detection: we generalized the set used for the moment method to a not necessarily simply connected set, and show that we can considerably improve particle detection in the presence of certain kinds of noise (e.g., salt and pepper noise, Figure 11) with this generalization. As a new analysis method for complex plasmas, in Section 5 we introduce image moments to get properties of the set of pixels belonging to a particle image. We show how to directly calculate simple geometric features (angle and length of a stripe) of a set of pixels using image moments. As a next step, invariant moments, such as central moments [36], Hu moments [36,46] or affine moments [36] might be used to discriminate particles from other disruptive features (e.g., laser reflection on parts of the plasma chamber) or even to discriminate different particle types (e.g., size or shape) from each other.

We present a postprocessing method in Section 6 to further enhance the accuracy of the detected particle positions by fitting a generalized Gaussian function to the intensity profiles of the particles. This is particularly interesting if prefiltering is necessary due to noisy images. Then, the postprocessing can reduce errors introduced by the prefilter (Figure 5 and Figure 8). Also, it can increase the sub-pixel resolution of particle positions. This is especially interesting for applications where small particle velocities, e.g., thermal velocities, are calculated from the positions (Section 7.1).

Another application is shown in Section 7.2: Particles which are close-by each other on the image plane can be separated by either manual or automatic threshold detection, and position accuracy was improved by the above postprocessing method. This kind of situation typically appears in the individual camera images of a stereoscopic imaging system.

In summary, image processing with a Hanning filter (alg03), and a subsequent blob detection with the moment method detects in the most cases all particles in our simulations, but needs a manually chosen threshold. Automatic threshold detection (alg04) results in a slightly reduced accuracy and a reduced detection rate, but has the advantage of the automatism. In both cases, postprocessing the acquired positions by fitting (alg06 and alg07) reduced uncertainties in the particle coordinates at the cost of a large calculation time (Figure 12 shows a factor of 60 to 144), but for specific experiments with the requirement of a good sub-pixel resolution this can be very useful and worth the effort.

## Figures and Tables

**Table 1:** This table shows a few examples of velocity errors in mm/s calculated from spatial resolution in mm/px and time resolution dt in ms. The pixel error results from our simulations (see Figure 8 and Figure 5) with the algorithms alg06 and alg07. Assuming a distribution around 0, the root mean square resulting from our simulations is the standard deviation. We neglect here that changing the spatial resolution also changes the size of a particle on the image sensor.

SNR	Pixel Error	Spatial Resolution	Time Resolution *dt*	Velocity Error
100	0.014	0.020	10	0.056
100	0.014	0.020	2	0.28
100	0.014	0.005	12.5	0.012

## Abbreviations

The following abbreviations are used in this manuscript:

PTV	Particle Tracking Velocimetry
PIV	Particle Image Velocimetry
SNR	Signal to Noise Ratio
e.g.	Exempli Gratia
alg01, alg02, *…*, alg07	different algorithms explained on page 10
alg08, alg09, alg10	different algorithms explained on page 12
alg11, alg12	different algorithms explained on page 18
